# Ubiquitin-mediated degradation at the Golgi apparatus

**DOI:** 10.3389/fmolb.2023.1197921

**Published:** 2023-07-06

**Authors:** Lana Buzuk, Doris Hellerschmied

**Affiliations:** Center of Medical Biotechnology, Faculty of Biology, University of Duisburg-Essen, Essen, Germany

**Keywords:** Golgi protein quality control, Golgi homeostasis, ubiquitin E3 ligase, transmembrane protein degradation, Golgi fragmentation, ubiquitin-mediated protein degradation, PROTAC

## Abstract

The Golgi apparatus is an essential organelle of the secretory pathway in eukaryotic cells. It processes secretory and transmembrane proteins and orchestrates their transport to other endomembrane compartments or the plasma membrane. The Golgi apparatus thereby shapes the cell surface, controlling cell polarity, cell-cell communication, and immune signaling. The cytosolic face of the Golgi hosts and regulates signaling cascades, impacting most notably the DNA damage response and mitosis. These essential functions strongly depend on Golgi protein homeostasis and Golgi integrity. Golgi fragmentation and consequent malfunction is associated with neurodegenerative diseases and certain cancer types. Recent studies provide first insight into the critical role of ubiquitin signaling in maintaining Golgi integrity and in Golgi protein quality control. Similar to well described pathways at the endoplasmic reticulum, ubiquitin-dependent degradation of non-native proteins prevents the accumulation of toxic protein aggregates at the Golgi. Moreover, ubiquitination regulates Golgi structural rearrangements in response to cellular stress. Advances in elucidating ubiquitination and degradation events at the Golgi are starting to paint a picture of the molecular machinery underlying Golgi (protein) homeostasis.

## Introduction

**FIGURE 1:**
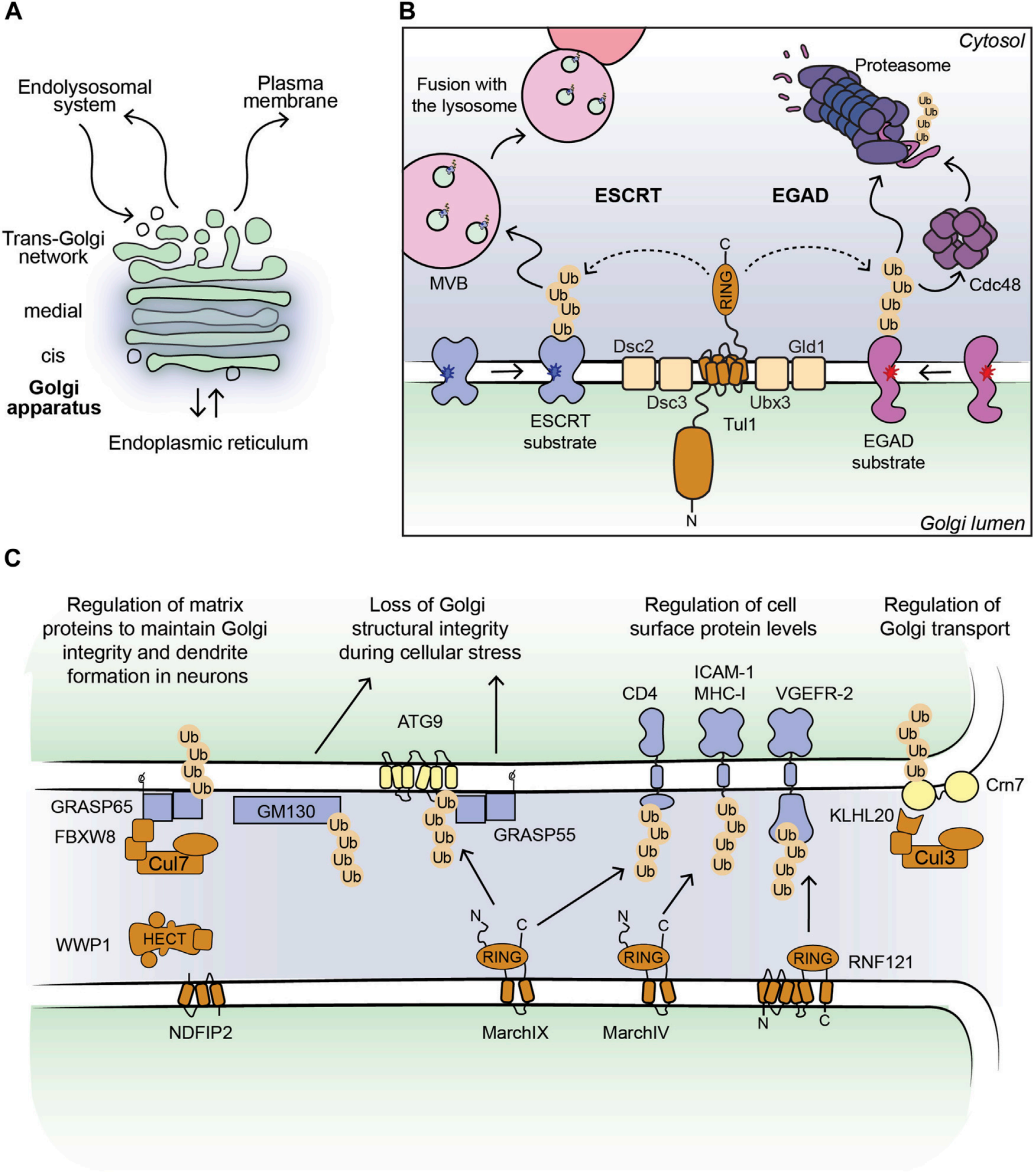
**(A)** Outline of a Golgi stack. **(B)** Cartoon model of the Dsc complex in yeast targeting substrates for ESCRT- and EGAD-mediated degradation. Non-native proteins recognized by the Dsc complex are shown in blue (ESCRT substrates) and purple (EGAD substrates). Dark blue and red stars indicate folding lesions within the TM domains of the substrate proteins. The Tul1 ubiquitinated ESCRT substrates are sorted into ILVs by the ESCRT machinery (not shown for simplicity). MVBs containing the ILVs fuse with the lysosome/vacuole and ubiquitinated proteins are degraded. The Tul1 ubiquitinated EGAD substrates are removed from the Golgi membrane and unfolded by Cdc48, and eventually degraded by the proteasome. **(C)** Current outline of E3 ligase-substrate pairs at the Golgi apparatus in mammalian cells. Selected E3 ligases (shown in brown) with their known substrates, where the ubiquitinated proteins can be targeted for degradation (shown in blue) or induce changes in protein-protein interactions (shown in yellow). The consequences of protein ubiquitination at the Golgi are stated in the text above.

Compartmentalization of eukaryotic cells creates unique membrane-enclosed reaction environments, organelles, with specialized functions. Together with the endoplasmic reticulum (ER), the Golgi apparatus forms the early secretory pathway in eukaryotic cells, responsible for the production and maturation of nearly all secretory and transmembrane (TM) proteins (Barlowe and Helenius, 2016; Barlowe and Miller, 2013). The Golgi apparatus links secretory and TM-protein maturation, mainly by glycosylation and protease processing of precursors, with transport to their correct cellular location (Ramazanov et al., 2021). This vital function is especially important for secretory cells and tissues, for example, anti-body producing plasma cells, pancreatic cells, and hepatocytes. To ensure the sequential modification of cargo proteins, modifying enzymes are segregated into distinct Golgi layers, which are arranged as polarized stacks (Lujan and Campelo, 2021) (Figure 1A). The intact Golgi stack further facilitates ordered cargo transport from cis-to-trans Golgi and sorting of cargo at the trans-Golgi network (TGN) into carriers targeted to the plasma membrane (PM) or the endo-lysosomal system (De Matteis and Luini, 2008; Chen et al., 2017; Lujan and Campelo, 2021). In mammalian cells, Golgi stacks are laterally linked into the Golgi ribbon, a critical scaffold for signaling cascades (Gosavi and Gleeson, 2017). Maintenance of the Golgi stack and ribbon is achieved by structural proteins and tethers, which constitute a protein-rich matrix on the cytosolic face of the Golgi (Xiang and Wang, 2011). Moreover, the cytoskeleton and small GTPases as well as their co-factors, which are involved in Golgi transport, regulate Golgi morphology (Kulkarni-Gosavi et al., 2019). Loss of Golgi integrity, Golgi fragmentation, is a hallmark of certain cancer types and an early phenotype in neurodegenerative disease (Joshi et al., 2015; Zhang, 2021; Spano and Colanzi, 2022). Molecular insight into (disturbed) Golgi homeostasis is therefore critical to our understanding of the pathophysiology of disease and may reveal therapeutic targets.

The essential functions of the Golgi apparatus are supported by Golgi-specific protein quality control (PQC) and homeostasis pathways (Sun and Brodsky, 2019; Benyair et al., 2022; Schwabl and Teis, 2022). The post-translational modification of proteins with ubiquitin is an important signal in these pathways. The ER- and Golgi-delimiting membranes provide an interface with the cytosolic ubiquitination machinery to impact on PQC processes and serve as a regulatory hub for organelle homeostasis signaling (Benyair et al., 2022; Krshnan et al., 2022; Rusilowicz-Jones et al., 2022; Schwabl and Teis, 2022). Within the secretory pathway, ubiquitination has been studied in great detail as part of the ER-associated degradation (ERAD) pathway, targeting misfolded proteins for proteasomal degradation (Sun and Brodsky, 2019). ERAD comprises the steps of substrate recognition, retrotranslocation to the cytosol and ubiquitination, shuttling to the proteasome and subsequent proteasomal degradation (Sun and Brodsky, 2019; Krshnan et al., 2022). The ubiquitination step requires a cascade of three enzymes. The E1 enzyme activates Ubiquitin (Ub) in an ATP-dependent manner and transfers it to the active site cysteine of an E2 enzyme (E2∼Ub) (Schulman and Harper, 2009). E3 ligases catalyze attaching Ub to the substrate protein, typically via isopeptide bond formation on lysine residues, and thereby confer substrate specificity to the system (Zheng and Shabek, 2017). Three major types of E3 ligases are distinguished based on their reaction mechanism–Really Interesting New Gene (RING)-type, homologous to E6AP C-terminus (HECT)-type, and RING-in-Between-RING (RBR)-type (Berndsen and Wolberger, 2014; Morreale and Walden, 2016). RING-type E3s activate E2∼Ub conjugates and promote the transfer of Ub from the E2 to the substrate protein (Berndsen and Wolberger, 2014; Morreale and Walden, 2016; Zheng and Shabek, 2017). HECT-type E3s form a thioester with Ub and directly transfer Ub to the substrate protein (Berndsen and Wolberger, 2014; Morreale and Walden, 2016; Zheng and Shabek, 2017). RBR-type E3s use a combination of the RING- and HECT-type mechanism (Berndsen and Wolberger, 2014; Morreale and Walden, 2016; Zheng and Shabek, 2017). They contain one RING domain recruiting the E2∼Ub conjugate and a second RING domain with a catalytic cysteine forming a thioester with Ub and transferring it to the substrate protein. E3 ligases can directly recognize their substrates or cooperate with substrate receptors, increasing the substrate spectrum of a given ligase. This concept is best illustrated by the largest group of E3s, the Cullin-RING ligase (CRL) family, where the Cullin subunit acts as scaffold for a RING domain protein and an interchangeable substrate adaptor/receptor pair (Rusnac and Zheng, 2020). In ERAD, E3 ligases collaborate with chaperones specifically supplying misfolded, non-native proteins from the ER lumen and membrane as substrate proteins (Krshnan et al., 2022).

Ub signals on substrates exist in the form of mono-Ub and different types of poly-Ub chains, with diverse downstream effects, a phenomenon referred to as the Ub code (Komander and Rape, 2012). In this review we focus on ubiquitin-mediated protein degradation as a downstream effect. Two major degradation systems exist in eukaryotic cells–lysosomes and the proteasome (Cohen-Kaplan et al., 2016; Bard et al., 2018). Proteasomal degradation of ubiquitinated substrates from cellular organelles requires substrate retrotranslocation to the cytosol, promoted by the AAA unfoldase p97 (Cdc48 in yeast). In ERAD, p97 extracts substrates from the ER membrane and interacts with so-called shuttling factors, coupling substrate retrotranslocation and proteasomal degradation (Christianson and Ye, 2014). Lysosomal degradation of ubiquitinated TM-proteins typically involves their transport to endosomes, followed by the formation of so-called multi-vesicular bodies (MVBs), which fuse with lysosomes (MacGurn et al., 2012; Migliano and Teis, 2018). Ubiquitin signals on organelles, possibly also at the Golgi, can serve as indictors of organelle stress and damage to recruit the cellular repair machinery or the autophagy machinery, which induces the degradation of damaged organelles (Harper et al., 2018; Papadopoulos et al., 2020)—a topic that will not be covered in this mini-review.

Recently ubiquitination cascades emerged as important pathways regulating proteins at the Golgi apparatus. Here we discuss our current understanding of ubiquitination and degradation events at the Golgi regulating PQC, Golgi morphology and transport processes. We focus on E3 ligases as the key factors selecting substrate proteins.

## The role of TM-ubiquitin ligases at the Golgi apparatus

E3 ligases targeting Golgi protein quality control substrates were initially reported and extensively studied in yeast (Reggiori and Pelham, 2002; Stewart et al., 2011; Schwabl and Teis, 2022). The yeast Tul1 RING-type E3 ligase is an integral membrane protein that localizes to the Golgi, endosomes, and the vacuole (Yang et al., 2018). Tul1 is part of the defective-for-SREBP-cleavage (Dsc) complex, a multi-protein complex with homology to the ER-localized Hrd1 complex, known for its prominent role in ERAD (Fonseca and Carvalho, 2019; Schmidt et al., 2019). Dsc subunits besides Tul1 are Dsc2, Dsc3, and Ubx3, which provides a docking site for the AAA unfoldase Cdc48 (Lloyd et al., 2013; Fonseca and Carvalho, 2019; Schmidt et al., 2019) (Figure 1B). Additional subunits that determine the sub-cellular localization of the Dsc complex are Vld1, inducing vacuolar targeting, and Gld1, supporting the cycling between Golgi and endosomes (Yang et al., 2018). The Dsc complex targets Golgi protein quality control (GQC) substrates for degradation. It ubiquitinates non-native proteins that contain, for example, polar residues within their TM-domains or unpalmitoylated proteins (Reggiori and Pelham, 2002; Valdez-Taubas and Pelham, 2005), and GQC model substrates, for example, GFP-Yif1 (Dobzinski et al., 2015). In addition to targeting non-native proteins for vacuolar degradation, Tul1 ubiquitinates vacuolar resident proteins Cps1 and Phm5 at the Golgi to initiate their transport to the vacuole (Reggiori and Pelham, 2002). Notably, not all ubiquitinated Tul1 substrates are targeted to the vacuole. Certain substrates follow the Endosome and Golgi-associated degradation (EGAD) pathway, which shows striking similarities to ERAD (Fonseca and Carvalho, 2019) (Figure 1B). For example, Orm2, a multi-pass TM-protein is ubiquitinated by Tul1, extracted from the Golgi membrane by Cdc48 and degraded by the proteasome (Schmidt et al., 2019). How proteins are triaged between the two proteolytic pathways is currently unknown. The versatile Tul1 also ubiquitinates the non-protein substrate phosphatidylethanolamine on endosomal and vacuolar membranes during starvation (Sakamaki et al., 2022). While mammalian cells lack a clear orthologue of Tul1, a handful of TM-proteins with a predicted cytosolic RING domain localize to the Golgi (Table 1) (Nakamura, 2011). Based on homology searches of the Tul1 RING domain, RNF122 and RNF24 may substitute for the Tul1 ubiquitination activity in humans (Schmidt et al., 2019). Both ligases were detected at the Golgi in mammalian cells (Table 1). However, RNF24 and -122 only have two predicted TM-domains, as opposed to seven in Tul1 and they lack a Golgi lumenal domain. While the role of the additional TM-domains and the large lumenal domain of Tul1 are currently unknown, RNF-24 and -122 cannot functionally replace them. Homologues of additional subunits of the Dsc complex in mammalian cells were predicted to be UBAC2 (Dsc2), TMUB1/2 (Dsc3), and UBXD8 (Ubx3), yet their role at the Golgi is largely elusive (Schmidt et al., 2019).

**TABLE 1:** Ubiquitin E3 ligases localized to the Golgi in mammalian cells.

E3 ligase	Uniprot ID	Size (aa)	Type of E3 ligase	Reported Golgi localization (other comments)	Reference(s)
RNF24	Q9Y225	148	Single-pass TM, RING-type	RING domain shows homology to Tul1, Golgi localization in SiHa cells (IF)	(Thul et al., 2017) (Schmidt et al., 2019)
RNF121	Q9H920	327	Multi-pass TM, RING-type (atypical)	Golgi localization in HEK293 cells (OE and imaging, Golgi-IP and mass spectrometry) and in A-431, U-251MG, U2OS, and HeLa cells (IF)	(Maghsoudlou et al., 2016) (Zemirli et al., 2014) (Fasimoye et al., 2022) (Thul et al., 2017)
				Substrate: VEGFR-2	
RNF122	Q9H9V4	155	Single-pass TM, RING-type	RING domain shows homology to Tul1, Golgi localization in HEK293 cells (OE and imaging)	(Schmidt et al., 2019) (Wang et al., 2006)
RNF125	Q96EQ8	232	Myristoylated, RING-type	Golgi localization in A-431 and SK-MEL-30 (IF)	Thul et al. (2017)
RNF149	Q8NC42	400	Single-pass TM, protease associated (PA) domain, RING-type	Golgi localization in HEK293 cells (proximity biotinylation)	Go et al. (2021)
RNF157	Q96PX1	679	Myristoylated, RING-type	Golgi localization in A-431, U-251MG, and U2OS cells (IF)	Thul et al. (2017)
RNF182	Q8N6D2	247	Multi-pass TM, RING-type	Golgi localization in HeLa cells (IF)	Thul et al. (2017)
RNF183	Q96D59	192	Single-pass TM, RING-type	Golgi localization in Cos7 and HeLa cells (OE and imaging)	(Wu et al., 2018a) (Wu et al., 2018b)
RNF214	Q8ND24	703	Coiled-coil domain, RING-type (atypical)	Golgi localization in A-431 and U-251MG cells (IF)	Thul et al. (2017)
MarchIV	Q9P2E8	410	Multi-pass TM, RING-CH	Golgi localization in HeLa cells (OE and imaging)	Bartee et al. (2004)
				Substrates: MHC-I, CD4	
MarchIX	Q86YJ5	346	Multi-pass TM, RING-CH	Golgi localization in HeLa cells (OE and imaging)	(Bartee et al., 2004) (Hoer et al., 2007) (Luo et al., 2022)
				Substrates: MHC-I, CD4, ICAM-1, ATG9	
DTX3	Q8N9I9	347	RING-type	Nuclear and Golgi-localized in Rh30 and U2OS cells (IF)	Thul et al. (2017)
ZFPL1	O95159	310	B-box, RING-type	Golgi localization in HEK293 cells (Golgi-IP and mass spectrometry) and in Vero, HeLa, A-431, U-251MG, and U2OS cells (IF) Interacts with GM130 at the Golgi	(Chiu et al., 2008) (Fasimoye et al., 2022) (Thul et al., 2017)
CBLC	Q9ULV8	474	Cbl-type phosphotyrosine-binding (Cbl-PTB) domain, RING-type	Binds to phosphorylated tyrosine residues, Golgi localization dependent on Src tyrosine kinase activity	Lee et al. (2015)
				Golgi-localized in HeLa cells (IF)	
CUL7-FBXW8	FBXW8 - Q8N3Y1	598	Cullin RING ligase complex	Golgi localization in mammalian granule neurons (FBXW8—by IF, Cul7—by OE and imaging) substrate protein: GRASP65, recruited to the Golgi by OBSL1, and possibly ARF1	Litterman et al. (2011)
CUL3-KLHL20	KLHL20 - Q9Y2M5	609	Cullin RING ligase complex	KLHL20 Golgi localization in Cos-1 cells (IF and presence in Golgi-enriched fractions) and in U-251MG (IF)	(Thul et al., 2017) (Li et al., 2022)
				Substrates: SERINC5, Crn7	
(CUL2)-FEM1A	FEM1A- Q9BSK4	669	Cullin-RING ligase complex	FEM1A Golgi localization in A431 cells (IF)	Thul et al. (2017)
WWP1	Q9H0M0	922	NEDD4 family, HECT-type	Golgi localization in A431 cells (IF)	Thul et al. (2017)
				Possible adaptor proteins for NEDD4 family ligases at the Golgi: NDFIP2 (Uniprot id: Q9NV92)—three TM-domains, PPxY motifs	

IF, immunofluorescence; OE, overexpression.

In mammalian cells, Golgi-localized multi-pass TM E3 ligases were shown to target proteins destined for the PM for degradation (Table 1; Figure 1C). Golgi E3 ligases thereby control the cell surface proteome responsible for cell-cell communication and immune signaling. The Golgi E3 RNF121, regulates the maturation of Vascular endothelial growth factor receptor 2 (VEGFR-2) (Maghsoudlou et al., 2016). Upon RNF121 knock-down, cell surface levels of VEGFR-2 increase, accompanied by a reduction of ubiquitinated VEGFR-2 (Maghsoudlou et al., 2016). Consistently, RNF121 over-expression leads to a decrease in mature VEGFR-2 levels and inhibits VEGF-induced cell proliferation and angiogenesis, revealing RNF121 as an important regulator of angiogenic growth factor signaling (Maghsoudlou et al., 2016). RNF121 was also identified as an essential host factor for infection by adenoviral vectors in a CRISPR KO screen and as an enhancer of NF-kB signaling in an siRNA screen (Zemirli et al., 2014; Madigan et al., 2019). Nevertheless, the role of RNF121-mediated ubiquitination in these pathways is so far unknown. Golgi-localized E3 ligases were also shown to target immune signaling proteins produced in the secretory pathway (Lin et al., 2019). The membrane-associated RING-CH (MARCH) family of E3 ligases comprises eleven members, initially discovered as homologues of the viral K3/K5 E3 ligase immune evasion proteins (Bartee et al., 2004; Lin et al., 2019). These proteins share a core domain architecture of a RING-CH domain (a RING domain coordinating Zn with a C4HC3 geometry) N-terminal to at least two TM-domains (Lin et al., 2019). MarchIV localizes to the Golgi and MarchIX to the TGN and to lysosomes (Bartee et al., 2004; Hoer et al., 2007). Reported substrates for both ligases are cell surface proteins MHC-I and CD4 (immune signaling molecules), and ICAM-1 (a cell adhesion protein) for MarchIX (Bartee et al., 2004; Hoer et al., 2007) (Figure 1C). The precise molecular events of substrate ubiquitination and degradation remain to be determined. Current data suggest that upon overexpression of MarchIV or MarchIX, MHC-I is ubiquitinated at its short cytosolic tail, endocytosed and degraded in lysosomes (Bartee et al., 2004). Upon heat stress, or inhibition of sialylation (a form of glycosylation at the Golgi), MarchIX also ubiquitinates ATG9, the only conserved TM component of the autophagy machinery at the TGN (Luo et al., 2022). Ubiquitination of ATG9 induces its interaction with the Golgi structural protein GRASP55, leading to a disruption of the Golgi matrix and Golgi fragmentation (Luo et al., 2022), highlighting a non-proteolytic role of ubiquitin signaling in controlling Golgi morphology during cellular stress. Another RING- and B-box domain containing protein, ZFPL1, localizes to the Golgi and directly interacts with Golgi matrix protein GM130 (Chiu et al., 2008; Fasimoye et al., 2022). While this interaction is critical for Golgi structural integrity (Chiu et al., 2008; Fasimoye et al., 2022), a role of ubiquitin signaling in this pathway remains to be determined.

## Soluble E3 ligases are recruited to Golgi substrate proteins

The concept of recruiting soluble E3 ligases to cellular membranes is well defined for yeast Rsp5, a HECT-type E3 with multiple substrates known at the PM, endosomes, and the Golgi (Sardana and Emr, 2021). Substrates or adaptor proteins containing a PPxY peptide motif bind Rsp5 and activate the E3 ligase at cellular membranes (Sardana and Emr, 2021). Rsp5 targets GQC substrates for vacuolar degradation (Wang et al., 2011), together with the PPxY-containing TM-adaptor Bsd2 (Hettema et al., 2004). Homologues of Rsp5 in mammalian cells belong to the Neuronal precursor cell-expressed developmentally downregulated 4 (NEDD4) family (Sardana and Emr, 2021). Among the NEDD4-type ligases, WWP1 localizes to the Golgi apparatus in certain mammalian cancer cells (Thul et al., 2017). Based on homology to yeast Bsd2 and its reported Golgi localization, NDFIP2 may recruit members of the NEDD4 family to the Golgi apparatus (Shearwin-Whyatt et al., 2004). A potential role of a NEDD4 ligase-adaptor complex in GQC remains to be determined.

In addition to NEDD4-type ligases, protein complexes of the CRL family are recruited to ubiquitinate Golgi substrates and thereby regulate Golgi morphology and Golgi transport processes. The levels of Golgi matrix proteins need to be tightly controlled, as both, depletion and overexpression compromise Golgi structural integrity, leading to defects in cell signaling cascades, protein modification, and protein sorting (Kulkarni-Gosavi et al., 2019). A complex of CUL7 with the substrate receptor FBXW8 regulates the turnover of GRASP65 to control Golgi structure and dendrite formation in neurons (Litterman et al., 2011). Knock-down of CUL7 or FBXW8 leads to GRASP65 accumulation, Golgi fragmentation, and reduced dendrite elaboration (Litterman et al., 2011). Co-depletion of CUL7-FBXW8 and GRASP65 abolishes the described phenotypic effects (Litterman et al., 2011). CRLs can also be recruited to target Golgi substrates for degradation using small molecule PROteolysis TArgeting Chimeras (PROTACs) and molecular glues (Diamantino et al., 2022). These molecules induce an interaction between E3 ligases and selected substrates, leading to substrate ubiquitination and subsequent degradation (Burslem and Crews, 2017). Multi-pass TM-proteins, solute carrier transporters, were targeted for degradation from the Golgi by fusion to the so-called dTAG domain and PROTAC-dependent recruitment of the Cullin-based CBRN E3 ligase (Bensimon et al., 2020). Moreover, Golgi matrix proteins GRASP55 and GRASP65, Golgi transport complex protein COG4, and the small GTPase RAB1 were fused to the auxin-inducible degron (AID) domain (Hatoyama et al., 2021; Zhang and Seemann, 2021; Sumya et al., 2023). Addition of auxin analogs, a type of molecular glue, recruits an ectopically expressed CRL inducing efficient and acute ubiquitination and proteasomal degradation of AID-fusion proteins (Burslem and Crews, 2017). Acute depletion of Golgi AID-fusion proteins revealed the role of GRASP-proteins in maintaining the Golgi ribbon (Zhang and Seemann, 2021) and showed that COG4 and RAB1 depletion compromises Golgi integrity, leading to Golgi protein mis-localization and a block in secretory transport, respectively (Hatoyama et al., 2021; Sumya et al., 2023). These examples highlight the power of targeted protein degradation strategies in inducing Golgi remodeling to impact Golgi function. Another CRL substrate receptor, FEM1A that works with CUL2-EloBC in targeting substrates with an exposed C-terminal arginine degron sequence for degradation, localizes to the Golgi (Thul et al., 2017; Koren et al., 2018). Golgi substrates of the complex are currently unknown. A molecular understanding of this complex at the Golgi may allow co-opting it for targeted degradation strategies.

Non-proteolytic roles for E3 ligases, where ubiquitination induces changes in protein-protein interaction networks at the Golgi were reported to regulate membrane trafficking. A CRL consisting of CUL3 and the KLHL20 substrate receptor localizes to the TGN and assembles K33-linked poly-Ub chains on its substrates (Yuan et al., 2014; Li et al., 2022). Poly-ubiquitination of the substrate Crn7 promotes Crn7 localization at the TGN, where it exerts its F-actin stabilizing activity to support transport carrier formation (Yuan et al., 2014). Disruption of this pathway leads to a block in basal secretory trafficking (Yuan et al., 2014). Poly-ubiquitination of Serinc5, an integral membrane protein, by CUL3-KLHL20 at the TGN promotes Serinc5 trafficking to the PM and thereby regulates its antiviral activity (Li et al., 2022). At the PM, Sercin5 is incorporated into HIV-1 viral particles and reduces their infectivity (Li et al., 2022). Golgi-related transport is also activated by phosphorylation, specifically by the tyrosine kinase Src (Pulvirenti et al., 2008). Src activity at the Golgi regulates recruitment of the CBLC E3 ligase, which maintains the Golgi ribbon (Lee et al., 2015). The CBLC RING domain is essential for its role in Golgi structural maintenance, however ubiquitinated Golgi substrate proteins have not yet been implicated (Lee et al., 2015). In addition to endogenous mammalian E3 ligases, enzymes encoded by bacterial pathogens also regulate the ubiquitination status of Golgi proteins during host cell infection to induce Golgi fragmentation and reduce the secretory capacity of the cell (Wan et al., 2019; Shin et al., 2020; Liu et al., 2021). This strategy may be aimed at immune evasion by reducing cell surface antigen display, but the precise physiological role remains to be determined.

## Degradation of ubiquitinated Golgi proteins

From the Golgi apparatus ubiquitinated proteins are targeted for degradation either by lysosomes or the proteasome. Lysosomal degradation is initiated by Golgi-to-endosome transport, a process mediated by Clathrin-coated vesicles and the GGA Clathrin adaptor proteins that bind to ubiquitinated substrates and sort them into transport vesicles (Pelham, 2004). At the endosome, the endosomal sorting complex required for transport (ESCRT) machinery captures ubiquitinated proteins and sorts them into MVBs by the continued generation of intralumenal vesicles (ILVs) (MacGurn et al., 2012; Migliano and Teis, 2018) Figure 1B. The ESCRT machinery is comprised of four sub-complexes: ESCRT-0, -I, -II and -III and an AAA-type ATPase, Vps4 (Vietri et al., 2020). ESCRT-0, -I, -II are responsible for binding and sorting the ubiquitinated proteins into ILVs. The membrane bending and scission activities of ESCRT-III and Vps4 respectively, lead to the release of the ILVs into the endosomal lumen. The resulting MVBs fuse with lysosomes where ILVs containing ubiquitinated proteins are degraded (Migliano and Teis, 2018; Vietri et al., 2020).

The EGAD pathway, discovered in yeast, targets ubiquitinated Golgi proteins for degradation by the proteasome, with the first identified substrate, Orm2 (Schmidt et al., 2019). Upon Orm2 ubiquitination by the Dsc complex at the Golgi, Cdc48 is recruited. Cdc48 typically engages ubiquitinated substrates in complex with its heterodimeric substrate adaptor Ufd1-Npl4 (Meyer and van den Boom, 2023). Moreover, the Dsc complex subunit Ubx3 contains a Cdc48-interacting UBX domain (Schmidt et al., 2019). The similarity of the Dsc complex to the ERAD Hrd1 complex suggests the requirement for a TM-channel for protein extraction and for shuttling factors targeting substrates to the proteasome. In analogy to EGAD, in mammalian cells the Golgi Apparatus-Related Degradation (GARD) pathway targets Golgi proteins for proteasomal degradation in response to stress (Eisenberg-Lerner et al., 2020). The Golgi matrix protein, GM130 is degraded upon treatment of cells with small molecules inhibiting sialyation or blocking intra-Golgi protein trafficking (Eisenberg-Lerner et al., 2020). The invoked stress on the Golgi induces the recruitment of p97 and proteasomes to Golgi membranes and causes the organelle to disassemble (Eisenberg-Lerner et al., 2020). The Golgi structure reverts to its pre-stress state upon wash-out of either treatment, showing how this mode of proteasome-dependent degradation sets out to maintain the general homeostasis of the Golgi (Benyair et al., 2022).

## Discussion

As part of PQC, ubiquitination at the Golgi targets non-native proteins for degradation to avoid the accumulation of potentially toxic protein species, which may disrupt Golgi integrity (Schwabl and Teis, 2022). Golgi PQC systems monitor Golgi resident proteins, but also cargo proteins that are continuously processed at the Golgi, ensuring the supply of functional cell surface and secretory proteins (Sun and Brodsky, 2019; Schwabl and Teis, 2022). To this end, the GARD and EGAD pathways operate at the Golgi in a similar manner as ERAD at the ER. The conceptual similarities support investigation of key substrate processing steps. The current molecular understanding of the Golgi PQC machinery stems mainly from work in yeast (MacGurn et al., 2012; Sardana and Emr, 2021; Schwabl and Teis, 2022). In mammalian cells, the molecular machinery for substrate recognition and ubiquitination at the Golgi still needs to be defined (Benyair et al., 2022; Schwabl and Teis, 2022). Moreover, in analogy to ERAD, the extraction of proteins from the Golgi membrane may require a yet-to-be-identified TM-channel and specific p97 co-factors. Finally, a key, Golgi-specific question to address is how ubiquitinated proteins are selected for proteasomal *versus* ESCRT-mediated lysosomal degradation. The fate of ubiquitinated Golgi proteins may be determined by the type of Ub signal assembled by the respective ubiquitination enzymes. Yet, work on Tul1 demonstrates that modification by the same ubiquitination complex may allow substrates to access more than one degradation pathway. This suggests an additional layer of regulation downstream of the ubiquitination event. To this end, Ub signals may be shaped by Golgi-localized deubiquitination enzymes (DUBs), Ub-specific proteases (Clague et al., 2019). It is also conceivable that the nature and accessibility of the substrate protein, or the kinetics of substrate processing determine the mechanism and/or outcome of Golgi protein degradation. Future work on Golgi PQC will resolve these exciting open questions. In addition to PQC, ubiquitination of Golgi proteins regulates Golgi homeostasis, specifically Golgi structural integrity and Golgi-related vesicle trafficking. Ubiquitination induces changes in Golgi morphology during stress by inducing Golgi matrix protein degradation and disrupting the matrix by changing protein-protein interaction networks, a strategy also adopted by bacterial effector proteins (Eisenberg-Lerner et al., 2020; Liu et al., 2021; Luo et al., 2022). These recent findings set the stage for an exciting new chapter in ubiquitin and Golgi research. Changes in Golgi morphology can compromise the cell’s secretory capacity but also signaling cascades that require the Golgi ribbon or stack as an assembly platform, as observed in different diseases (Gosavi and Gleeson, 2017; Makhoul et al., 2019; Spano and Colanzi, 2022). On the other hand, stress-induced Golgi morphology changes are found to be reversible, suggesting a dynamic process acting in favor of eventually restoring homeostasis. Defining the substrate spectrum of Golgi E3 ligases (Table 1) and their activity profile in different cell types, during cellular stress, and in disease will lead to a better understanding of ubiquitination in Golgi homeostasis. In the long term, new insights may support the development of strategies, which co-opt (Golgi) E3 ligases, such as PROTACs, aimed at controlling the structure and accordingly the function of the Golgi apparatus.

## Figures and Tables

**Table:** 

**ER**	endoplasmic reticulum
**TM**	Transmembrane
**TGN**	Trans-Golgi network
**PM**	Plasma membrane
**PQC**	Protein quality control
**ERAD**	ER-associated degradation
**Ub**	Ubiquitin
**RING**	Really Interesting New Gene
**HECT**	Homologous to E6AP C-terminus
**RBR**	RING-in-Between-RING
**CRL**	Cullin-RING ligase
**MVBs**	Multivesicular bodies
**GQC**	Golgi protein quality control
**Dsc**	Defective-for-SREBP-cleavage
**Tul1**	Transmembrane E3 ubiquitin-protein ligase 1
**Hrd1**	HMG-CoA reductase degradation protein 1
**Ubx3**	Ubiquitin regulatory X UBX domain-containing protein 3
**Cdc48**	Cell division control protein 48
**Vld1**	Vacuole localized Dsc protein 1
**Gld1**	Golgi localized Dsc protein 1
**Cps1**	Copalyl disphosphate synthase 1
**Phm5**	Phomasetin biosynthesis cluster protein 5
**EGAD**	Endosome and Golgi-associated degradation
**RNF**	RING finger
**UBAC2**	Ubiquitin-associated domain-containing protein 2
**TMUB1/2**	Transmembrane and ubiquitin-like domain containing protein 1/2
**UBXD8**	Ubiquitin-X domain adaptor 8
**VEGFR-2**	Vascular endothelial growth factor receptor 2
**CRISPR KO**	Clustered Regularly Interspaced Short Palindromic Repeats knockout
**MARCH**	Membrane-associated RING-CH
**MHC-I**	Major histocompatibility complex-I
**CD4**	Cluster of differentiation 4
**ICAM-1**	Intercellular adhesion molecule 1
**ATG9**	Autophagy-related gene 9
**GRASP55**	Golgi reassembly-stacking protein of 55 kDa
**ZFPL1**	Zinc finger protein-like 1
**GM130**	Golgi matrix protein 130
**Rsp5**	Reverses SPT-phenotype protein 5
**NEDD4**	Neuronal precursor cell-expressed developmentally downregulated 4
**Bsd2**	Bypass superoxide dismutase defects protein 2
**NDFIP2**	NEDD4 family interacting protein 2
**WWP1**	WW domain-containing protein 1
**FBXW8**	F-box/WD repeat-containing protein 8
**GRASP65**	Golgi reassembly-stacking protein of 65 kDa
**CUL7**	Cullin-7
**PROTACs**	PROteolysis TArgeting Chimeras
**CBRN**	Cereblon
**RAB1**	Ras-related protein Rab-1A
**AID**	Auxin-inducible degron
**COG4**	Conserved oligomeric Golgi complex subunit 4
**FEM1A**	Protein fem-1 homolog A
**EloBC**	Elongin B/Elongin C
**CUL3**	Cullin-3
**KLHL20**	Kelch-like protein 20
**Crn7**	Coronin-7
**CBLC**	Casitas B-lineage lymphoma proto-oncogene c
**GGA**	Golgi-localized, gamma-ear containing, ADP-ribosylation factor binding
**ESCRT**	Endosomal sorting complex required for transport
**ILVs**	Intralumenal vesicles
**Vps4**	Vacuolar protein sorting-associated protein 4
**Ufd1**	Ubiquitin fusion degradation protein 1
**Npl4**	Nuclear protein localization protein 4 homolog
**GARD**	Golgi Apparatus-Related Degradation
